# Remarkable Reduction of Cocaine Use in Dual Disorder (Adult Attention Deficit Hyperactive Disorder/Cocaine Use Disorder) Patients Treated with Medications for ADHD

**DOI:** 10.3390/ijerph16203911

**Published:** 2019-10-15

**Authors:** Corrado Manni, Giada Cipollone, Alessandro Pallucchini, Angelo G. I. Maremmani, Giulio Perugi, Icro Maremmani

**Affiliations:** 1 School of Psychiatry, University of Pisa, 56100 Pisa, Italy; corrado3@live.it (C.M.); cipollonegiada@gmail.com (G.C.); pallucchini.a@gmail.com (A.P.); 2 Department of Psychiatry, North-Western Tuscany Region NHS Local Health Unit, Versilia Zone, 55049 Viareggio, Italy; angelo.maremmani@uslnordovest.toscana.it; 3 Association for the Application of Neuroscientific Knowledge to Social Aims (AU-CNS), 55045 Pietrasanta, Lucca, Italy; 4 G. De Lisio Institute of Behavioral Sciences, 56100 Pisa, Italy; 5 Second Psychiatric Unit, Department of Clinical and Experimental Medicine, University of Pisa, 56100 Pisa, Italy; giulio.perugi@med.unipi.it; 6 Vincent P. Dole Dual Disorder Unit, Santa Chiara University Hospital, University of Pisa, 56100 Pisa, Italy

**Keywords:** cocaine use disorder, stimulant medication, atomoxetine, methylphenidate, recovery from cocaine dependence

## Abstract

*Background*: Cocaine use disorder (CUD) is a growing public health concern, but so far no effective pharmacotherapies have been demonstrated. Stimulant medications have proved to be promising in CUD treatment. The self-medication hypothesis (SMH) can help to explain this phenomenon better, especially in cases where CUD co-occurs with adult attention deficit hyperactivity disorder (A-ADHD). *Methods*: In the present retrospective study, a sample of 20 consecutive patients (aged from 18 to 65 years) with dual disorder (A-ADHD/CUD), under treatment with methylphenidate (MPH) or atomoxetine (ATM) medications, was followed to study the effects of A-ADHD treatment on cocaine use. Patients were followed for a mean period of 7 months (minimum 1, maximum 30 months). All individuals were assessed with standardized questionnaires to evaluate diagnosis, treatment efficacy, and clinical improvement. *Results*: the results showed that behaviors reflecting cocaine addiction were sharply reduced during the stimulant treatment of A-ADHD, and were not correlated with age, gender, familiarity, length of treatment, or medication used. CUD improvement was closely correlated with the A-ADHD improvement. This study supports the validity of the SMH in ADHD patients with co-occurring CUD.

## 1. Introduction

Cocaine use disorder (CUD) is a growing public health concern. As estimated in the annual World Drug Report for 2018 of the United Nations Office for Drugs and Crime (WDR 2018), in 2016, the number of past-year cocaine users increased globally by almost 7% from the previous year to 18.2 million (range: 13.9–22.9 million). More than half of all cocaine users live in the Americas, mostly North America (34% of the global total). Almost one-quarter of the world total for users reside in Europe, especially in Western and Central Europe, which alone account for about one-fifth of the total number. Africa and, to a lesser extent, Asia and Oceania, taken together, may account for the remaining quarter of all cocaine users, although these estimates are biased by a significant lack of data for these geographical areas.

These figures for all age groups include about 2.3 million young adults aged 15–34 (1.9% of this age group) who have used the drug in the last year. Over the past few decades, the role of attention deficit hyperactivity disorder (ADHD) in the development and persistence of problematic substance behaviours up to the addictive level has been given extensive clinical attention, while studies have shown an increasing prevalence of ADHD (mean = 23.1%; 95% CI = 19.4–27.2%) [1], covering a range of different substance use disorders (SUDs). Moreover, the most recent data demonstrate that leaving ADHD untreated has a negative impact on SUD treatment outcomes. Greater efforts are now being made to discover effective pharmacological strategies for ADHD in comorbidity with SUD, but recent findings remain inconclusive [2,3].

To better explain the association between ADHD and SUD, primarily focusing on cocaine use disorder (CUD), the self-medication hypothesis (SMH) could help. The drug SMH was developed by Khantzian, who suggested that the drive to take drugs may be thought of by the user as the best practical solution to relieve suffering and distress, especially with reference to heroin and cocaine.

It has been suggested that there is a considerable degree of psychopharmacological specificity in the selected used substances [4,5]. The drugs that addicts select are not chosen randomly but are the end result of an interaction between the psychopharmacological action of the drug and psychiatric symptoms causing compelling distress, compounded with the factors of proneness and vulnerability. Subjects self-select which substance to use according to their personality structure and related impairments. 

Consequently, as stated by the SMH, patients use drugs as a way to suppress ADHD symptoms. Stimulants including cocaine, can, however paradoxically, act to calm and counteract hyperactivity, emotional lability, and inattention in ADHD individuals [6].

Similarly, Holtmann et al. [7] and Gudjonsson et al. [8] reported an increased likelihood of alcohol, cannabinoid, heroin, and cocaine use in ADHD adolescents to relieve the specific psychopathological features derived from this disorder, namely affective dysregulation, implying an overall vulnerability to a difficult mental state, which is marked by susceptibility to boredom, need for stimulation, and distress associated with cognitive performance.

However, the data made available so far are inadequate and inconclusive, often considering SUD overall regardless of the specific type of drug, and are mostly limited to childhood and late adolescence. One major drawback has been that little is known about ADHD and CUD in adulthood.

Aims: In the present retrospective case series study, a sample of 20 consecutive patients (age range 18−65) with adult ADHD (A-ADHD) and co-occurring CUD, under treatment with methylphenidate (MPH) or atomoxetine (ATM), was followed for a mean period of 7 months (minimum 1, maximum 30 months) to study the effects of ADHD treatment on cocaine use.

## 2. Materials and Methods

### 2.1. Design of the Study

Research information on patients included in the present study came from the A-ADHD Clinic (A-ADHD outpatient clinic) study, a cohort study including information on individual patients admitted to stimulant medication treatment for A-ADHD at the Second Psychiatric Unit of Santa Chiara University Hospital, University of Pisa, Italy. For the aims of the present study, data collected at the time of enrolment into the study (baseline) and at the last available evaluation, invariably including the presence of a condition of cocaine use in each patient, were extracted from the A-ADHD-Clinic dataset. Extracted information was analyzed following a cross-sectional design to estimate the magnitude of differences between patients in terms of the clinical global impression of A-ADHD and addictive behavior (amount of cocaine used). 

All the subjects examined signed an informed consensus document that allowed them to participate in the A-ADHD-Clinic study. Both the consent form and the experimental procedures were approved by the ethics committee of the University of Pisa (study ID: 14003; code: ADHD-MOOD), in accordance with internationally accepted criteria for ethical research. Aims, data gathered, and the future perspectives of this case series study are summarized in Table 1.

### 2.2. Sample

All patients had to satisfy the following inclusion criteria:

- Not having been diagnosed in childhood/adolescence
- First diagnosis of ADHD in adulthood
- Comorbidity of substance use disorder, with patient choice of cocaine as the primary substance of use.
- Treatment for almost a month.
- Compliance with treatment confirmed by a family informant
- Not under treatment with the combination of atomoxetine (ATM)/metylphenidate (MPH)

All patients had received stable and comparable psychopharmacological treatment during the whole observational period, except for stimulants. Patients with current psychotic symptoms, hypomanic or manic states, and cardiovascular comorbidities were excluded.

The selected sample included 20 subjects. Eighteen (90.0%) were males, 16 (80.0%) were single, and all had had an education lasting at least eight years. Six (30.0%) were white-collar workers, 2 (10.0%) were blue-collar workers, and 12 (60.0%) were unemployed. Eighteen (90.0%) reported an adequate economic level. Only three (15.0%) were living alone. Age was 30 ± 11 years (range: 16−61).

### 2.3. Assessment

Both A-ADHD and the CUD diagnosis were made with the Structured Clinical Interview for DSM-5 (SCID-5), following DSM-5 criteria. A diagnosis of ADHD was at first investigated with the A-ADHD Self-Report Scale (ASRS) screening test and confirmed by using the Structured Diagnostic Interview for A-ADHD (DIVA). All individuals were assessed with standardized questionnaires to evaluate treatment efficacy and clinical improvement (Clinical Global Impression, CGI) and cocaine addiction severity (Cocaine Problem Severity Index, CPSI).

#### 2.3.1. Structured Clinical Interview for DSM-5 (SCID-5)

The Structured Clinical Interview for DSM-5 (SCID-5) [9] is a semi-structured interview guide to making the primary DSM-5 diagnoses. It has to be administered by a clinician or trained mental health professional who is familiar with the DSM-5 classification and its diagnostic criteria. The interview subjects may be either psychiatric or general medical patients, or individuals who do not identify themselves as patients, such as participants in a community survey of mental illness or family members of psychiatric patients. 

#### 2.3.2. Adult ADHD Self-Report Scale (ASRS-v1.1)

This scale is a self-reported instrument consisting of the 18 DSM−5 criteria. Six of the 18 questions were found to be those that best predicted ADHD symptoms. These six questions are the basis for the ASRS v1. Part B of the Symptom Checklist contains the remaining 12 questions [10]. We used the Italian version [11].

#### 2.3.3. Diagnostic Interview for ADHD in Adults (DIVA)

The DIVA is the first structured Dutch interview for ADHD in adults; it was developed by Kooij and Francken and is the successor of the earlier Semi-Structured Interview for ADHD in adults [12]. In order to simplify the evaluation of each of the 18 symptom criteria for ADHD, both in childhood and adulthood, the interview provides a list of concrete and realistic examples, for both current and retrospective (childhood) behaviour. The examples are based on the descriptions most often provided by adult patients in clinical practice. Examples are also given to investigate the types of impairment found in five areas of everyday life: work and education; relationships and family life; social contacts; free time and hobbies; self-confidence and self-image.

Whenever possible, the DIVA should be completed by the psychiatrist face to face with an adult patient in the presence of a partner and/or family member, to promote the provision of retrospective and collateral information. The DIVA usually takes around one and a half hours to complete. The DIVA only asks about the core symptoms of ADHD that are required for the formulation of a diagnosis of ADHD, regardless of co-occurring psychiatric disorders. The DIVA is divided into three parts, each applied both to childhood and adulthood: the criteria for attention deficit (A1), the criteria for hyperactivity–impulsivity (A2), and age at onset and impairment. Information received from the partner and immediate family is mainly meant to supplement the information obtained from the patient, and so provide the psychiatrist with an accurate account of both current and childhood behaviour. Caregiver information is particularly useful for childhood, since many patients usually struggle to recall their behaviour retrospectively. For each criterion, the researcher should decide its presence or absence in both the main stages of life, taking into account the information obtained from all the parties involved. If collateral information cannot be made available, the diagnosis should be based on the patient’s own recollection alone. If school reports are available, these can help to give an idea of the symptoms noticed in the classroom and can be used to support the diagnosis. Symptoms are considered clinically relevant when they occur at a more severe degree, more frequently than in the peer group, and inducing greater impairment of the individual’s skills. We used the Italian version [13].

#### 2.3.4. Clinical Global Impression (CGI)

Clinical Global Impression is a questionnaire designed by Guy [14]. It is a three-item observer-rated scale that measures the severity of illness (CGI-S), global improvement or change (CGI-C), and therapeutic response or efficacy (CGI-E). CGI-S is rated on a seven-point scale using a range of responses from 1 (normal) to 7 (among the most severely ill patients). The CGI-C scores range from 1 (very much improved) through to 7 (very much worsened). CGI-E is rated from 1 (vast improvement with no side effects) to 16 (unchanged or worsened, with side effects outweighing the therapeutic effects), by making assessments both for therapeutic efficacy and treatment-related adverse events at the same time. 

#### 2.3.5. Cocaine Problem Severity Index (CPSI)

The Cocaine Problem Severity Index (CPSI) is a test designed to help determine the extent of patients’ involvement with cocaine [15]. The test comprises 17 questions, offering a choice between four mutually exclusive answers. The CPSI considers: (1) the length of cocaine use, (2) the frequency of use; (3) the type of cocaine use; (4) the quantity of use; (5) the longest period of abstinence during the previous 12 months; (6) the presence of depressive symptoms/craving; and (7) other somatic symptoms appearing once cocaine use has stopped. Patients must also specify: (8) the frequency of use of other drugs taken; (9) the frequency of alcohol use, (10) difficulties experienced at work arising from cocaine use; (11) relationship difficulties caused by cocaine use; (12) household difficulties caused by cocaine use; (13) financial difficulties caused by cocaine use; and (14) the differences arising from cocaine use in sexual activities. Two questions are related to: (15) cocaine or (16) alcohol use by friends, and (17) how the patient gets his/her supply of cocaine. The last item listed (18) reports four conditions that require immediate assistance. The sum of all these items indicates the severity of the patient’s involvement with cocaine.

### 2.4. Procedure

The assessments were scheduled at baseline and every month (±15 days) for the first three months, then at intervals of three months (±1 month). The CGI was administered using A-ADHD and CUD by two researchers who were mutually blinded and also blinded with respect to the previous evaluations. Control over bias from different evaluators was partially ensured by the supervision of a senior psychiatrist (I. Maremmani). 

### 2.5. Data Analysis

The baseline/last-evaluation differences for each of the A-ADHD and CUD CGI-S, CGI-C, CGI-E, and CPSI questionnaire items and for their total were tested by the Wilcoxon signed-rank test, which was used to compare a paired difference in a single sample when the population could not be assumed to be normally distributed.

The correlations between the CGI-S, CGI-C, and CGI-E at the last evaluation of ADHD and CUD were calculated with partial correlation analysis using Pearson’s *r* corrected by baseline values. We compared, using the Mann–Whitney test (the non-parametric test for independent samples), last-evaluation ADHD-CUD CGI-S, CGI-C, and CGI-E with a set of subgroups (males/females, above/below the mean age, short-term/long-term treated, polysubstance user/no-polysubstance user) to test whether our results were correlated with them.

We used Statistical routines of SPSS (v25.0) (IBM, Armonk, NY, USA) to elaborate our data. 

## 3. Results

### 3.1. Clinical Characteristics of the Sample at Treatment Entry

ADHD combined with a predominantly inattentive presentation was the main finding in three (15.0%) patients. All patients met the psychiatric comorbidity criteria for bipolar 1 disorder. Regarding the co-presence of other SUDs, eight (40.0%) patients were affected by cannabis use disorder; six (30.0%) by opioid use disorder (OUD) in agonist opioid treatment (AOT); one (5.0%) by alcohol use disorder (AUD); and two (10.0%) by hallucinogen use disorder. We followed up with 12 patients (60.0%) for less than 6 months and with eight (40.0%) for more than 6 months (minimum 1, maximum 30). We treated 11 (55.0) patients with MPH (nine patients with MPH extended release and two patients with MPH immediate release) and nine (45.0%) with ATM. Patients were also treated with standard doses of ADHD medication, adding valproic acid and low dosages of benzodiazepines according to the stage of their bipolar illness. In Italy the standard dose of ATM is 1.2 mg/kg/day, the maximum dosage of immediate release MPH is 60 mg/day, and the standard dose of extended release MPH is 60–90 mg/day.

Regarding family history, five (25.0%) patients did not show psychiatric or SUD familiarity. Nine (45.0%) patients showed both psychiatric and SUD familiarity. Six (30.0%) showed only a psychiatric comorbidity. 

Regarding ADHD, after evaluation by the CGI-S, one patient (5.0%) was mildly ill, three (15.0%) were moderately ill, six (30.0%) were markedly ill, eight (40.0%) were severely ill, and two (10.0%) were among the most extremely ill patients. Moreover, regarding CUD, three (15.0%) were moderately ill, seven (35.0%) were markedly ill, seven (35.0%) were severely ill, and three (15.0%) were among the most extremely ill patients we had observed in our entire clinical practice.

As to the length of use, all patients had been cocaine-dependent for more than one year and nine (45.0%) for more than five years. Regarding the degree of severity, according to the CPSI total score, 19 (95.0%) patients were classified as affected by “cocaine dependence requiring assistance”. In this kind of patient, cocaine abuse is generally a serious problem. One patient was in the phase of “severe dependence”. In general, with these patients, it may be difficult for them to regain control of their life without hospitalization.

### 3.2. End-Point/Baseline Changes Regarding CPSI and CGI Related to ADHD and CUD

Table 2 reports changes in the CPSI-Questionnaire. All patients obtained an improvement (negative rankings) on the CPSI total score. On the question of the frequency of cocaine use at baseline, only one patient (5.0%) used cocaine once a week or less. Ten (50.0%) used cocaine 4−6 times per week and nine (45.0%) every day. At end point, all our patients reported use of “once a week or less”.

Regarding the other CPSI items, none of our patients showed a severity worsening (positive ranking), with the exception of two patients who increased their alcohol use, two who worsened their working condition, and one who improved despite more use of cocaine in his peer group.

In passing from baseline to end point, 80.0% of our patients improved, 5.0% worsened, and 15.0% showed an unchanged ADHD-CGI severity index (*z* = −3.603 *p* < 0.001). Ninety percent of our patients improved, and 10.0% showed no change in the CUD-CGI severity index (*z* = −3.764; *p* < 0.001).

The efficacy index of ADHD-CGI radically improved in three patients (15.0%), much improved in eight patients (40.0%), and only minimally improved in nine patients (45.0%). None of them showed any side effects that could have interfered with their functioning. 

The efficacy index of CUD-CGI turned out to have greatly improved in four patients (20.0%), much improved in 10 patients (50.0%), and minimally improved in six patients (30.0%). None of them showed any side effects that could have interfered with their functional skills.

### 3.3. Relationship between CGI Outcomes and Other Clinical Aspects

**Figure 1:**
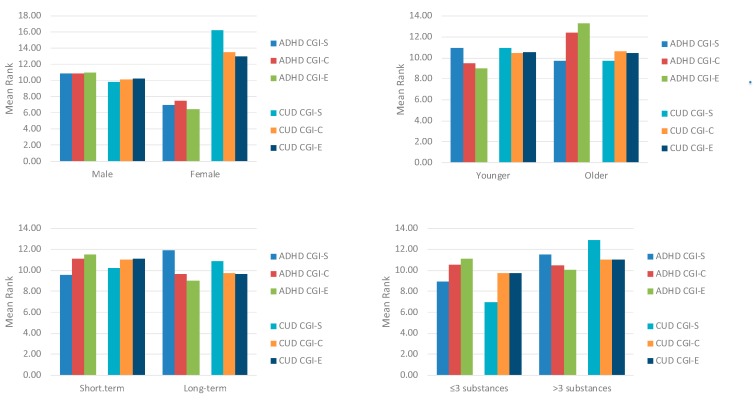
Relationship between CGI outcomes and other clinical aspects; CGI = Clinical Global Impression; ADHD CGI-S = Attention Deficit Hyperactive Disorder Severity of illness; ADHD CGI-C = Attention Deficit Hyperactive Disorder Change; ADHD CGI-E = Attention Deficit Hyperactive Disorder Efficacy; CUD CGI-S = Cocaine Use Disorder Severity of illness; CUD CGI-C = Cocaine Use Disorder Change.

Figure 1 reports the relationship between CGI outcomes and other clinical aspects. Last-evaluation ADHD-CGI-S, ADHD -CGI-C, and ADHD-CGI-E were not correlated with gender (*z* = −0.97, *p* = 0.442; *z* = −0.80, *p* = 0.516; *z* = −1.08, *p* = 0.379, respectively), younger/older age (*z* = −0.48, *p* = 0.699; *z* = −1.13, *p* = 0.257; *z* = −1.66, *p* = 0.096, respectively), short-term/long-term treatment (*z* = −0.94, *p* = 0.427; *z* = −0.57, *p* = 0.624; *z* = −0.99, *p* = 0.384, respectively), and baseline polysubstance use (*z* = −1.07, *p* = 0.343; *z* = −0.04, *p* = 0.970; *z* = −0.41, *p* = 0.734, respectively). Last-evaluation CUD-CGI-S, CUD-CGI-C, and CUD-CGI-E were not correlated with gender (z = −1.55, *p* = 0.120; z = −0.82, *p* = 0.516; *z* = −0.69, *p* = 0.589, respectively), younger/older age (*z* = −0.46, *p* = 0.669; *z* = −0.08, *p* = 0.931; *z* = −0.04, *p* = 0.965, respectively), or short-term/long-term treatment (*z* = −0.24, *p* = 0.851; *z* = −0.50, *p* = 0.678; *z* = −0.59, *p* = 0.624, respectively). CUD-CGI-S was higher in polyusers (*z* = −2.35, *p* = 0.025), while CUD-CGI-C and CUD-CGI-E did not differ statistically (*z* = −0.50, *p* = 0.678; *z* = −0.51, *p* = 0.678, respectively).

CGI outcomes were also not correlated with MPH/ATM use (ADHD-CGI-S: *z* = −1.68 *p* = 0.092; ADHD-CGI-C: *z* = −1.61, *p* = 0.872; ADHD-CGI-E: *z* = −0.28, *p* = 0.774; CUD-CGI-S: *z* = −0.04 *p* = 0.968; CUD-CGI-C: *z* = −0.66, *p* = 0.508; CUD-CGI-E: *z* = −0.75, *p* = 0.480).

ADHD (*z* = −1.01, *p* = 0.309) and CUD (*z* = −1.80, *p* = 0.071) CGI-C showed no statistically significant differences between patients with and without psychiatric or SUD familiarity. 

Table 3 reports partial correlations between ADHD and CUD last-evaluation CGI-S, CGI-C, and CGI-E corrected by the baseline ADHD-CUD-S. The CGI-C of the two illnesses was directly correlated independently of the baseline severity. The higher the improvement in patients’ ADHD, the greater the decrease recorded in cocaine use. CGI-S and CGI-E were not correlated.

## 4. Discussion

The patients we observed had an A-ADHD severity compatible with outpatient treatment, but their cocaine use was medium to high, and the sample even included patients in a severe condition. Regardless of the medication used, the reduction in cocaine use was important; it was also independent of gender, age, familiarity, duration of treatment, and concomitant use of other substances. This cocaine use reduction was directly correlated with the improvement in patients’ A-ADHD.

In the literature, there are no conclusive data demonstrating the efficacy of stimulant treatments in ADHD patients with SUD comorbidity. Treatment with stimulant medications has already proved to be effective in treating ADHD symptomatology, but only a few, still controversial findings attest to the efficacy of stimulant and non-stimulant medications on SUD in patients with concomitant ADHD [16].

As shown by Schubiner et al. [17], long-acting stimulants and non-stimulants (e.g., ATM) could become effective treatments with a lower risk of abuse potential. No other studies, however, have confirmed this hypothesis. For example, a single-site, randomized, controlled trial was conducted on 70 adolescents (aged 13–19) to evaluate the effects of ATM versus placebo on ADHD combined with SUD [18]. Another study by Konstenius was conducted on 54 participants who had a co-diagnosis of ADHD and amphetamine dependence and had been recruited from medium-security prisons in Sweden [19]. All patients were given MPH with doses up to 180 mg/day to treat ADHD and prevent any relapse into drug abuse. The MPH-treated group showed a reduction in their ADHD symptoms during the trial (*p* = 0.011) and had a significantly higher proportion of drug-negative urine samples compared to the placebo group (*p* = 0.047), including more amphetamine-negative urine samples (*p* = 0.019) and better retention in treatment (*p* = 0.032).

An earlier double-blind study compared MPH, bupropion, and placebo [20]. No advantages of drugs versus placebo were reported in reducing cocaine use; in addition, no differences were detected between the two drugs.

Some controlled randomized trials with short- and long-acting standard doses of MPH showed no improvement in SUD symptoms [17,20,21]. In another study, robust doses of extended-release, mixed amphetamines (60 and 80 mg/day) led to substantial reductions in both ADHD and drug consumption in cocaine-dependent patients with ADHD [22].

Therefore, while comparing our results to these controversial data, our clinical impression is that the dramatic reduction of CUD symptoms in patients with concomitant ADHD might be due to the specific craving investigated in our subjects. According to Khantzian’s theory, all of them may use stimulant drugs to reduce their ADHD inattentive symptoms, aiming for an improvement in their attention as well as relief from their restlessness. Thus, we might do better to define this desire for cocaine as a craving for relief from the cognitive and emotional distress arising from ADHD, which leads to severe impairment of the quality of life while drastically compromising social and professional functionality. This type of craving is different from the type usually reported by cocaine users who are free from ADHD, as the latter are mainly driven by the recreational and rewarding effects induced by the stimulant. In one of our studies we demonstrated that bipolar patients can use stimulants during the hypomanic or manic phase for rewarding reasons [23], but in our patients the presence of a hypomanic or manic state was not present at treatment entry nor did it appear during treatment. In bipolar 1 A-ADHD/CUD patients, stimulant medications seem to improve both ADHD and CUD by acting on the mental distress caused by an untreated ADHD, thus lowering the urgent need for relief which patients otherwise try to achieve through cocaine. It follows that the use of cocaine may work as self-medication behaviour, as previously proposed by Khantzian, in subjects who feel less interest in pleasurable feelings and reward than non-CUD individuals who have ADHD.

Moreover, we detected no significant differences in effectiveness between ATM and MPH, whether evaluated by clinicians or patients, through CGI and CPSI, respectively. Hence, the two different medications seem to exert similar positive influences on cocaine addiction in terms of severity and long-term outcomes. These results are not influenced by patients’ familiarity.

According to the results of our study, we propose the following pathophysiological model: subjects with A-ADHD, when taking cocaine, can reduce their symptoms of inattention and, therefore, implement this “relief craving” by continuing to consume the substance. Over time, cocaine, which is fast-acting, has a dysphoric but addictive action. So, the two diseases could be said to sum their respective negative effects; in any case, we should bear in mind that the initially strong euphoric effect of cocaine is amply exceeded by the dysphoric effect brought into play by the intoxication/withdrawal syndrome. When therapeutic stimulants, which take a long time to become effective and therefore have no dysphoric effect, reduce inattentiveness, the “need” for cocaine is reduced in proportion to the reduction of the inattentive symptoms that are induced by the ADHD disease, and of the dysphoric symptoms induced by cocaine addiction.

The clinical implications of this hypothesis include the possibility of identifying this type of subject at the beginning of patients’ drug addiction practice and in the real world. At our first interview with them, all our patients reported an action of cocaine on themselves other than that observed in their peers, because they felt less excited and more active. It is therefore essential to investigate the rewarding or relieving action of cocaine at the beginning of the drug addiction experience, both to deliver a diagnosis of A-ADHA as early as possible and to prevent cocaine dependence by treating ADHD with the kinetics of slow-acting stimulants. In our sample, the use of cocaine did not stop completely and tended to remain occasional. Previous study found that adults treated with MPH resulted in significant increases in post- vs. pre-treatment heart rate and systolic blood pressure as compared to placebo [24]. Combination with cocaine will further increase heart rate and blood pressure, leading to cardiotoxicity. This use of cocaine could be stimulated by the rewarding action of the substance that is no longer necessary for relief but would still carry out a rewarding action because of its kinetics. In other words, we would move from self-therapeutic to recreational use.

More specifically, we postulate that the use of extended-release (ER) medications such as MPH ER, and ATM could offer an effective treatment for these patients, reproducing the same effect as cocaine, but with slower kinetics. Consequently, greater tonic dopamine release due to these dopamine agonists might prevent relapses in CUD patients, in a way similar to the action of opioid agonists such as methadone and buprenorphine (long-acting agonists) in heroin (a fast-acting drug) use disorder. This study indirectly confirms the SMH in A-ADHD patients with co-occurring CUD.

Limitations: This study had some limits. The small sample size is the first to be mentioned. Consequently, further studies are needed to effectively confirm our hypothesis by increasing the number of enrolled patients. Secondly, this is a retrospective rather than a double-blind study. In exploring the comparison between two different treatments, MPH and ATM, our data might have been influenced by the factor of having two different stimulants available for use in treating each patient. It could have occurred that patients with less severe ADHD were treated with ATM, while MPH was set aside to treat the most severe cases.

## 5. Conclusions

Our findings suggest that the pharmacological therapy for DD A-ADHD/CUD patients is not only useful in improving inattention and hyperactivity but could also have a positive impact on cocaine-use prognosis and outcome by reducing the use of cocaine from intense to occasional. In addition, these data are not in contrast to Khantzian’s self-medication hypothesis.

## Figures and Tables

**Table 1:** Aims, data gathered, and future perspectives.

**Aims**
● To study the time-effects of attention deficit hyperactivity disorder (ADHD) treatment on cocaine use ● To support the self-medication model of this dual disorder
**Data gathered**
● Demographics ⚬ Age ⚬ Gender ⚬ Length of treatment ⚬ Presence of poly-substance use (>3) ⚬ Methylphenidate (MPH)/atomoxetine (ATM) use ● Diagnostic evaluation ⚬ Structured Clinical Interview for Diagnostic and Statistic Manual-5 (DSM-5), (SCID-5) ⚬ Adult ADHD Self-Report Scale (ASRS) ⚬ Diagnostic Interview for ADHD in Adults (DIVA) ● Clinical evaluation ⚬ Clinical Global Impression (CGI) severity (CGI-S) ⚬ CGI improvement or change (CGI-C) ⚬ CGI efficacy (CGI-E) ⚬ Cocaine Problem Severity Index (CPSI)
**Future perspectives**
⚬ To improve the knowledge about a subgroup Cocaine Use Disorder (CUD) response in ATM/MPH patients ⚬ Confirmatory clinical trials

**Table 2:** Changes in the CPSI questionnaire and CGI severity index related to ADHD and CUD (Wilcoxon signed-rank test).

	Negative Rankings T1 < T0	Positive Rankings T1 > T0	Ties T1 = T0	*z*	Two-Tailed *p*
CPSI-Questionnaire					
1-Length of use					
2-Frequency of use	19 (95.0%)	0 (0.0%)	1 (5.0%)	−3.904	0.000
3-Type of cocaine use	9 (45.0%)	0 (0.0%)	11(55.0%)	−2.80	0.005
4-Quantity of use	17 (85.0%)	0 (0.0%)	3 (15.0%)	−3.72	0.000
5-Longest consecutive period in the last year	13 (65.0%)	0 (0.0%)	7 (35.0%)	−3.241	0.001
6-Depressive symptoms/craving when having stopped cocaine use	12 (60.0%)	0 (0.0%)	8 (40.0%)	−3.093	0.002
7-Other somatic symptoms after having stopped cocaine use	10 (50.0%)	0 (0.0%)	10 (50.0%)	−2.844	0.004
8-Other drugs: frequency of use	9 (45.0%)	0 (0.0%)	11 (55.0%)	−2.682	0.007
9-Alcohol: frequency of use	8 (40.0%)	2 (10.0%)	10 (50.0%)	−2.339	0.019
10-Working difficulties due to cocaine use	8 (40.0%)	2 (10.0%)	10 (50.0%)	−2.339	0.019
11-Relationship difficulties due to cocaine use	13 (65.0%)	0 (0.0%)	7 (35.0%)	−3.220	0.001
12-Household difficulties due to cocaine use	14 (70.0%)	0 (0.0%)	6 (30.0%)	−3.376	0.001
13-Financial difficulties due to cocaine use	14 (70.0%)	0 (0.0%)	6 30.0%)	−3.345	0.001
14-Importance of cocaine in sexual activities	12 (60.0%)	0 (0.0%)	8 (40.0%)	−3.108	0.002
15-Friends using cocaine	12 (60.0%)	1 (5.0%)	7 (35.0%)	−2.977	0.003
16-Friends using alcohol	12 (60.0%)	0 (0.0%)	8 (40.0%)	−3.097	0.002
17-Cocaine: supply of	16 (80.0%)	0 (0.0%)	4 (20.0%)	−3.704	0.000
18-Severity	17 (85.0%)	0 (0.0%)	3 (15.0%)	−1.604	0.109
Total CPSI	20 (100.0%)	0 (0.0%)	0 (0.0%)	−3.921	0.000

T0 = baseline; T1 = endpoint; z = z test of Wilcoxon signed-rank test.

**Table 3:** Partial correlations* between ADHD-CUD last-evaluation CGI indexes corrected by the baseline ADHD-CUD severity index.

	Last-Evaluation ADHD CGI-S	Last-Evaluation ADHD CGI-C	Last-Evaluation ADHD CGI-E
Last-evaluation CUD CGI-S	*r* = 0.381 *p* = 0.119		
Last-evaluation CUD CGI-C		*r* = 0.503 *p* = 0.033	
Last-evaluation CUD CGI-E			*r* = 0.283 *p* = 0.255

* = Partial correlation corrected by baseline values; *r* = Pearson’s *r*; ADHD CGI-S = Attention Deficit Hyperactive Disorder Severity of illness; ADHD CGI-C = Attention Deficit Hyperactive Disorder Change; ADHD CGI-E = Attention Deficit Hyperactive Disorder Efficacy; CUD CGI-S = Cocaine Use Disorder Severity of illness; CUD CGI-C = Cocaine Use Disorder Change; CUD CGI-E = Cocaine Use Disorder Efficacy.
